# Microbiological analyses of nasally guided catheters after less invasive surfactant administration – a pilot study

**DOI:** 10.1186/s12887-020-02147-0

**Published:** 2020-05-19

**Authors:** Christian A. Maiwald, Julia Dick, Matthias Marschal, Christian Gille, Axel R. Franz, Christian F. Poets

**Affiliations:** 1 Department of Neonatology, University Children’s Hospital Tübingen, Tübingen, Germany; 2 Center for Pediatric Clinical Studies (CPCS), University Children’s Hospital Tübingen, Tübingen, Germany; 3 Institute of Medical Microbiology and Hygiene, University Hospital Tübingen, Tübingen, Germany

**Keywords:** LISA, MIST, Neonate, Respiratory distress syndrome, Surfactant administration

## Abstract

**Background:**

Respiratory distress syndrome (RDS) is a frequent complication of premature birth. Treating RDS by continuous positive airway pressure and less invasive surfactant administration (LISA) may reduce bronchopulmonary dysplasia. Surfactant, however, can be inactivated by bacterial infection. Therefore, potential routes of microbe transmission into the airway are of interest. The aim of this study was to evaluate microbiological contamination of catheters used for LISA procedures and its association with postnatal age.

**Methods:**

Catheter tips used for LISA procedures via the nasal route (LISA-n) in infants with RDS were placed into a sterile eSwab container directly after the procedure, cultured and examined for microbiological contamination.

**Results:**

Interpretable results could be collected from 20 catheter tips. Four showed positive culture results (20%) with microbes potentially associated with the development of early onset neonatal sepsis. Risk of positive microbe detection increased with postnatal age (< 4 h: 10%; 4–18 h: 20%; > 18 h: 40%).

**Conclusions:**

In this pilot study, the risk of tracheal microbe transmission following the LISA-n procedure increased with postnatal age. Although the clinical relevance of this finding is unclear, earlier surfactant administration might reduce the risk of catheter contamination.

**Trial registration number:**

Substudy of the registered Trial: feasibility study – Neofact: NCT04086095, www.ClinicalTrials.gov, September 11, 2019.

## Background

Pulmonary morbidity is a key issue in the treatment of premature infants. In the first postnatal days, respiratory distress syndrome (RDS) is, next to early-onset infection, one of the most important and common diseases affecting the lung.

Recent evidence suggests that RDS is best treated by continuous positive airway pressure (CPAP) combined with less invasive surfactant administration (LISA, [1–3]). Most reports using this approach administered surfactant via a thin catheter (TCA, [3–6]); this was also favored in a recent update of the European Consensus Guidelines on the Management of Respiratory Distress Syndrome [4].

Although most LISA reports and recently developed LISA catheters, such as LisaCath [5] or Neofact (formerly QuickSF [6]) prefer the oral route for insertion, the use of a nasally guided, soft umbilical vein catheter (LISA-n) is quite common in Germany. This involves the theoretical risk of catheter contamination with bacteria residing in the upper respiratory tract. It is unclear, however, how common this occurs in practice. We thus set out to quantify this risk in infants with RDS treated via LISA-n.

Main objective of this study was to evaluate microbiological contamination of the catheters used in LISA-n procedures and its association with postnatal age.

## Methods

### Ethical conditions

The study protocol was approved by the Ethics committee of University Hospital Tübingen. Samples were cultured anonymously (only numbered by order of occurrence). Parents gave written consent to their baby’s study participation.

### Collection of samples

We planned to collect 20 catheter tips from infants with RDS (gestational age, 23 + 0/7–36 + 0/7 weeks) and a clinical indication of surfactant administration following the LISA-n procedure. Catheters used were umbilical vein catheters with a diameter of 2.5 to 3.5 French. Following surfactant administration, catheters were removed and the tip (~ 1-3 cm) was cut with sterile scissors and directly placed in a sterile eSwab container with 1 ml liquid Amies solution (Copan Diagnostics, Murrieta, CA). After that, they were sent anonymously (only labelled with the study title and the next consecutive number) to the microbiology laboratory for culture; this happened immediately after the procedure if occurring on weekdays between 8 am and 8 pm, or following storage in a refrigerator at 4–8 °C till the next morning. Room temperature was allowed for no more than 2 h; also, if storage exceeded 24 h, the sample was discarded.

A consignment note to each sample collected the details of the infant’s medical history (e.g. postnatal age at the time of the procedure, number of surfactant administrations, etc.) and remained on the ward. Anonymization of the consignment note took place after reaching 48 h of postnatal age.

### Microbiological procedures

Specimens were thoroughly vortexed and 10 μl of Amies medium diluted in 990 μl sterile sodium chloride solution (0.9%). 100 μl of the undiluted sample and 100 μl of the dilution were then cultured for 48 h at 36 ± 1 °C on the following solid media: Columbia sheep blood agar (Oxoid, Thermo Fisher Diagnostics, Wesel, Germany) and Endo agar supplemented with 1% fuchsine (Oxoid) under aerobic conditions, plus brain heart infusion agar (Oxoid) with 5% sheep blood and IsoVitaleX enrichment (BD, Sparks, MD) under anaerobic conditions. Agar dishes were checked for microbial growth after 24 and 48 h.

In case of growth, colony forming units (CFU) were counted and microbes identified based on morphology, hemolysis, catalase and/or DNase production, as well as Staph Plus Latex Kit (DiaMondiaL, Sees, France).

### Statistical analysis

All analyses were descriptive and results were grouped by postnatal age: < 4 h, 4–18 h, > 18 h.

## Results

25 catheter-tips were collected between September 2018 and June 2019. Infants included into the study had a mean gestational age of 28 weeks (range 24–33 weeks). Baseline characteristics of the included infants, timing of the surfactant administrations, collection of samples and microbiological findings are shown in Table 1.

**Table 1:** Baseline characteristics of included infants, timing of surfactant doses, collection of samples and microbiological findings

No.	Gestational age at birth in weeks	Weight at birth in gram	Time of surfactant administration after birth in hours: minutes *cultured sample; *e collected, but excluded; ^--^ missed			Microbiological findings Bacterium (Colony Forming Units (CFU))
			1st dose	2nd dose	3rd dose	
1	24 + 5/7	345	00:13 ^--^	09:09 *	–	**viridans group streptococci (160 CFU)**
2	33 + 2/7	1730	34:23 *	–	–	**–**
3	29 + 0/7	1190	05:30 *	–	–	**–**
4	29 + 6/7	1600	00:22 ^--^	17:22 *	–	**–**
5	25 + 0/7	660	02:10 *e	17:50 *	–	**–**
6	27 + 2/7	980	01:02 *	–	–	**–**
7	31 + 4/7	2145	00:12 *	–	–	**–**
8	28 + 6/7	1340	01:49 *	–	–	**–**
9	31 + 2/7	1490	00:20 ^--^	02:38 *	18:53 ^--^	**–**
10	33 + 1/7	1990	25:21 *	–	–	**viridans group streptococci (160 CFU) coagulase-negative staphylococci (90 CFU)**
11	30 + 0/7	980	33:58 *	–	–	**coagulase-negative staphylococci (30 CFU)**
12	30 + 2/7	1220	00:08 *	–	–	**–**
13	26 + 6/7	655	26:21 *	–	–	**–**
14	25 + 4/7	495	02:24 *	37:09 ^--^	–	**–**
15	25 + 2/7	685	00:43 *	–	–	***Staphylococcus aureus*(60 CFU)**
16	29 + 5/7	1380	02:56 *	–	–	**–**
17	24 + 6/7	880	01:14 *	16:12 *e	–	**–**
18	25 + 4/7	555	01:03 ^--^	14:20 *	–	**–**
19	27 + 1/7	585	00:27 *	–	–	**–**
20	30 + 0/7	780	14:21 ^--^	17:55 *e	21:40 *	**–**

LISA-n was performed from 1 to a maximum of 3 times (overall mean: 1.5 times; 1.3 times in positive samples and 1.6 times in negative samples). Mean postnatal age at sample collection was 11.0 h (range: 0.1–34.4 h); it was 9.4 h (range: 0.1–34.4) in those with negative, and 17.3 h (range: 0.7–34.0 h) in those with positive microbiological culture results.

Five catheter tips had to be excluded because storage time had been > 24 h or they came from an infant already enrolled. Of the remaining 20 catheter tips, 4 showed a positive culture result (20%, see Table 1).

**Fig. 1:**
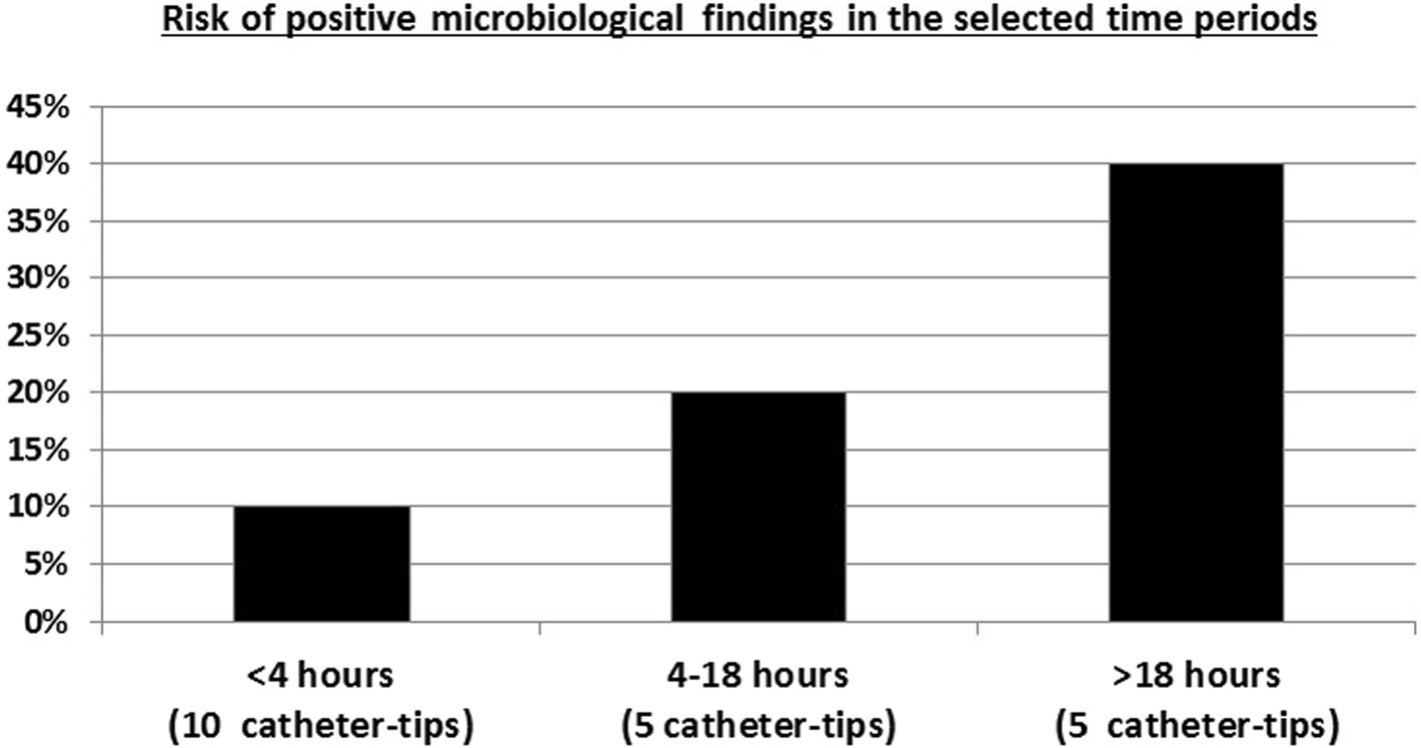
Risk of positive microbiological findings in the selected time periods

The calculated risk of catheter contamination increased with postnatal age (see Fig. 1).

## Discussion

We found a 20% risk of bacterial contamination for nasally introduced catheters, which increased with postnatal age. All bacterial species identified are known to be potentially associated with the development of early onset neonatal sepsis [7]. We do not know, however, whether our findings would have any effect on clinical outcome. Since differentiation of radiological signs of pneumonia and RDS is difficult and biochemical and clinical signs of sepsis might be affected by other reasons (e.g. prenatal infection that could have caused premature birth) a much higher sample size would be required to assign such findings to the surfactant administration procedure.

Results of this study are limited by the fact that it remains unclear whether the bacteria from positive catheter tips had already colonized the trachea [8] or whether colonization took place during catheter removal, which would then not involve a risk of transmission. This would have required an additional intratracheal smear before implementation of the LISA-catheter, which was regarded too invasive.

However, colonization of the catheter tip took place during its pathway through the upper respiratory tract (including the trachea) and by this there is a potential risk of microbe transmission when a LISA procedure is performed via the nasal route, as is probably also true for any nasally introduced endotracheal tube. Additionally, the risk of positive microbial culture results increased with postnatal age. This was expected owing to the fact that higher postnatal age will be accompanied with a higher rate of bacterial colonization of the pharynx and gastrointestinal tract.

Surfactant administration occurring immediately after birth is known to achieve a better effect in reducing air leaks than later surfactant administration. It also decreases the risk of pneumothorax, pulmonary interstitial emphysema, neonatal mortality and chronic lung disease in comparison to later rescue surfactant [9]. Additionally, late surfactant administration may also increase the risk of patent ductus arteriosus [10]. An increasing risk of bacterial catheter contamination as shown here adds another argument why early identification of infants requiring exogenous surfactant is important.

## Conclusion

It could be hypothesized that there is a potential risk of microbial transmission to the trachea of neonates in LISA-n procedures with higher postnatal age. However, it remains unclear if the findings of this study reflect the risk of transmission or colonization of the trachea. If there is a risk of microbe transmission, it is also unknown if orally guided catheters during LISA or other TCA procedures would minimize this risk. Earlier administration of surfactant after birth would reduce this risk, thus early identification of infants who require surfactant would be important.

Further studies will be needed to determine if these findings have any effect on clinical outcome.

## Data Availability

The datasets used and/or analysed during the current study are available from the corresponding author on reasonable request.

## Abbreviations

CPAP: Continuous positive airway pressure
LISA: Less invasive surfactant administration
LISA-n: Less invasive surfactant administration nasally guided catheter
RDS: Respiratory distress syndrome
TCA: Thin catheter administration
